# Supplementary material for: Widespread contribution of transposable elements to the rewiring of mammalian 3D genomes
Source: Nat Commun. 2023 Feb 6;14:634. doi: 10.1038/s41467-023-36364-9 (PMC9902604; doi:10.1038/s41467-023-36364-9)
Supplement: Supplementary file 1 — Supplementary Information [file 41467_2023_36364_MOESM1_ESM.pdf]

Supplementary Information

Supplementary Figure 1: Data Set Resolution compared with TE Contribution to Loop Anchors

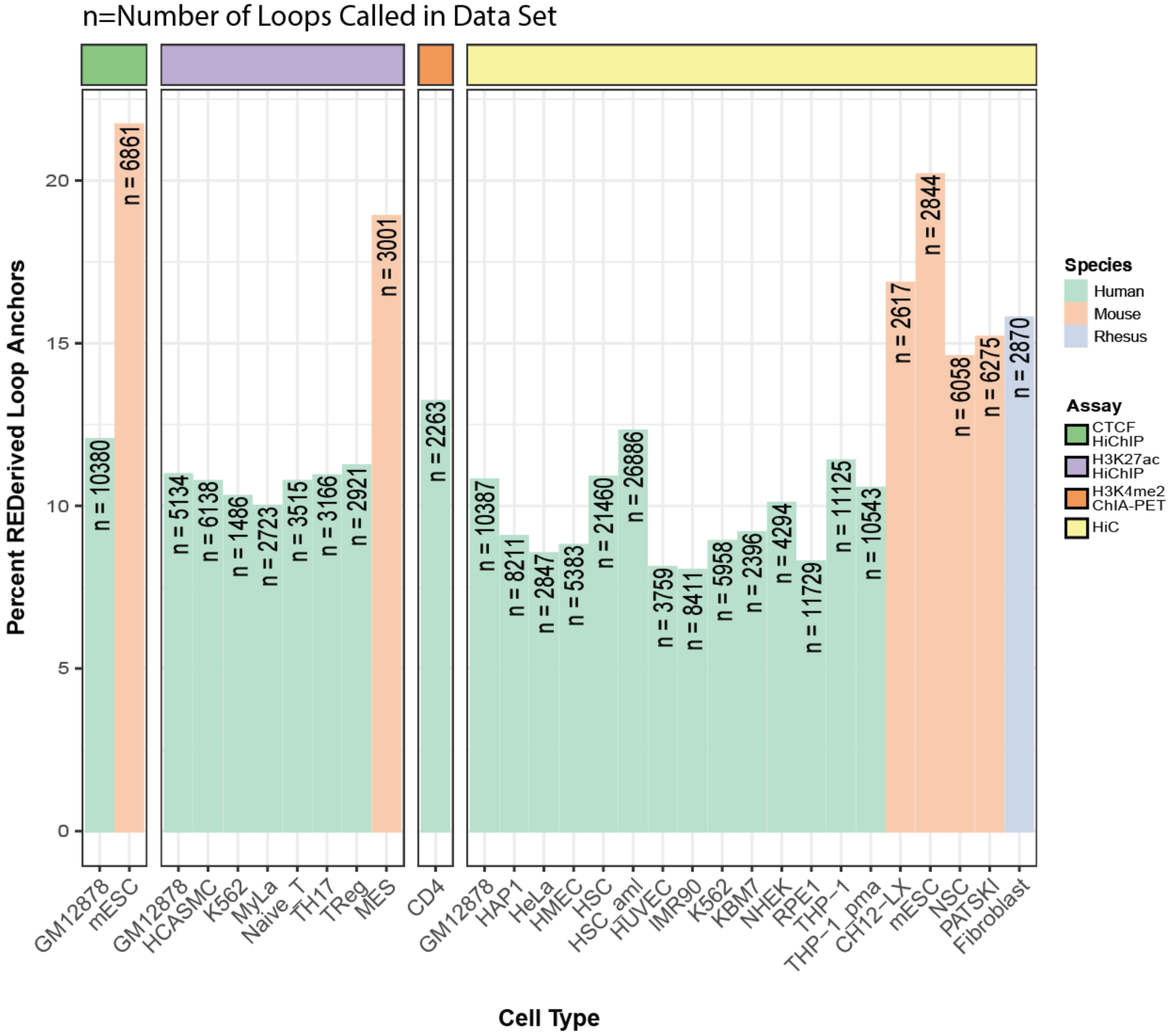

The number of loops called in each dataset is displayed in each of the corresponding bars. The y-axis shows the percent of loop anchors that overlap a repetitive element for each of the included datasets.

Supplementary Figure 2: Shuffled RE contribution to 3D Organization

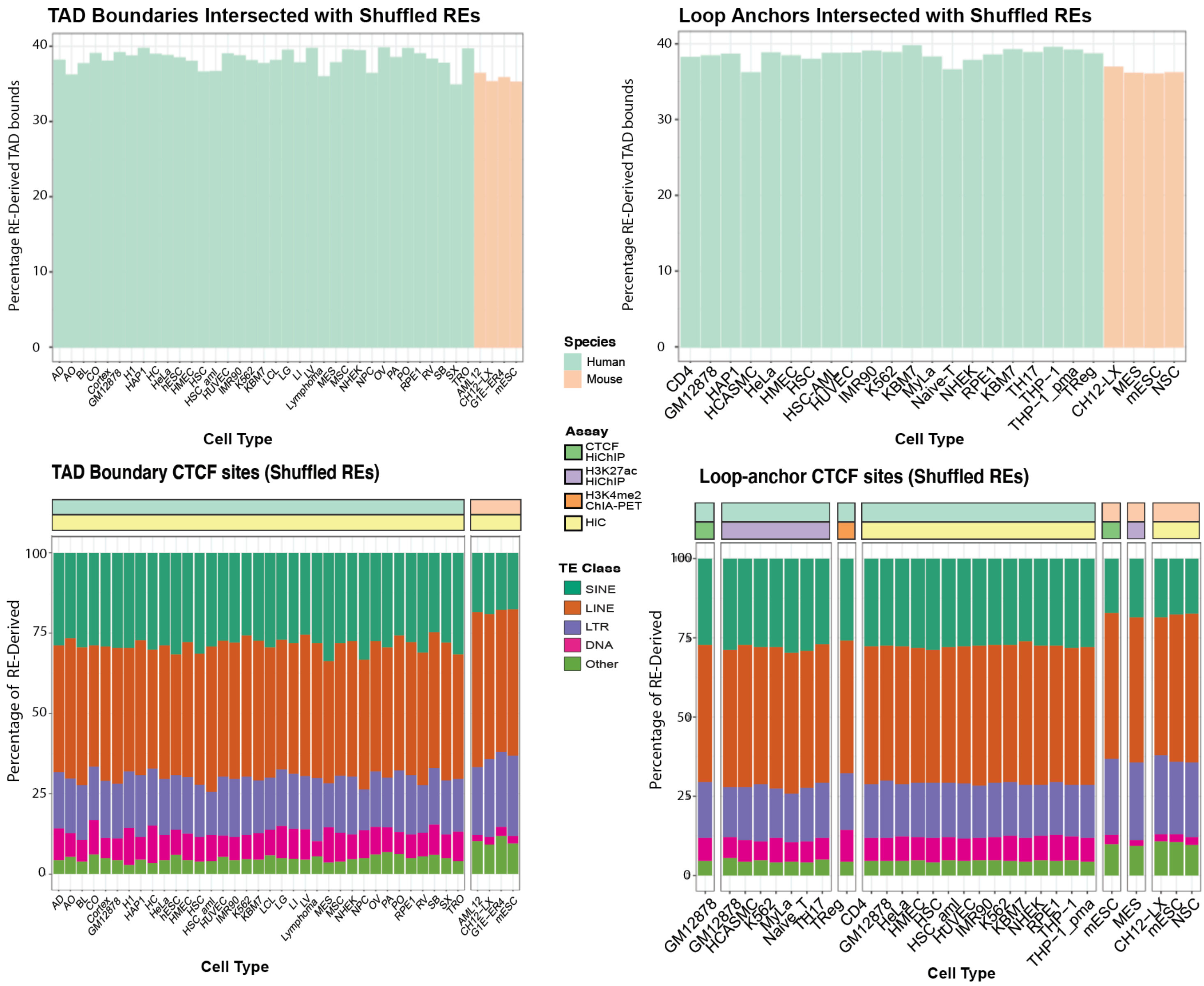

Hg19 and mm9 RepeatMasker files were shuffled using bedtools and these shuffled RE locations were intersected with loop and TAD boundaries to find the rate of RE-derived loop anchors and TAD boundaries that would occur if REs were randomly distributed in the genome.

Supplementary Figure 3: Comparison of Human and Dog Liver CTCF TAD Boundaries

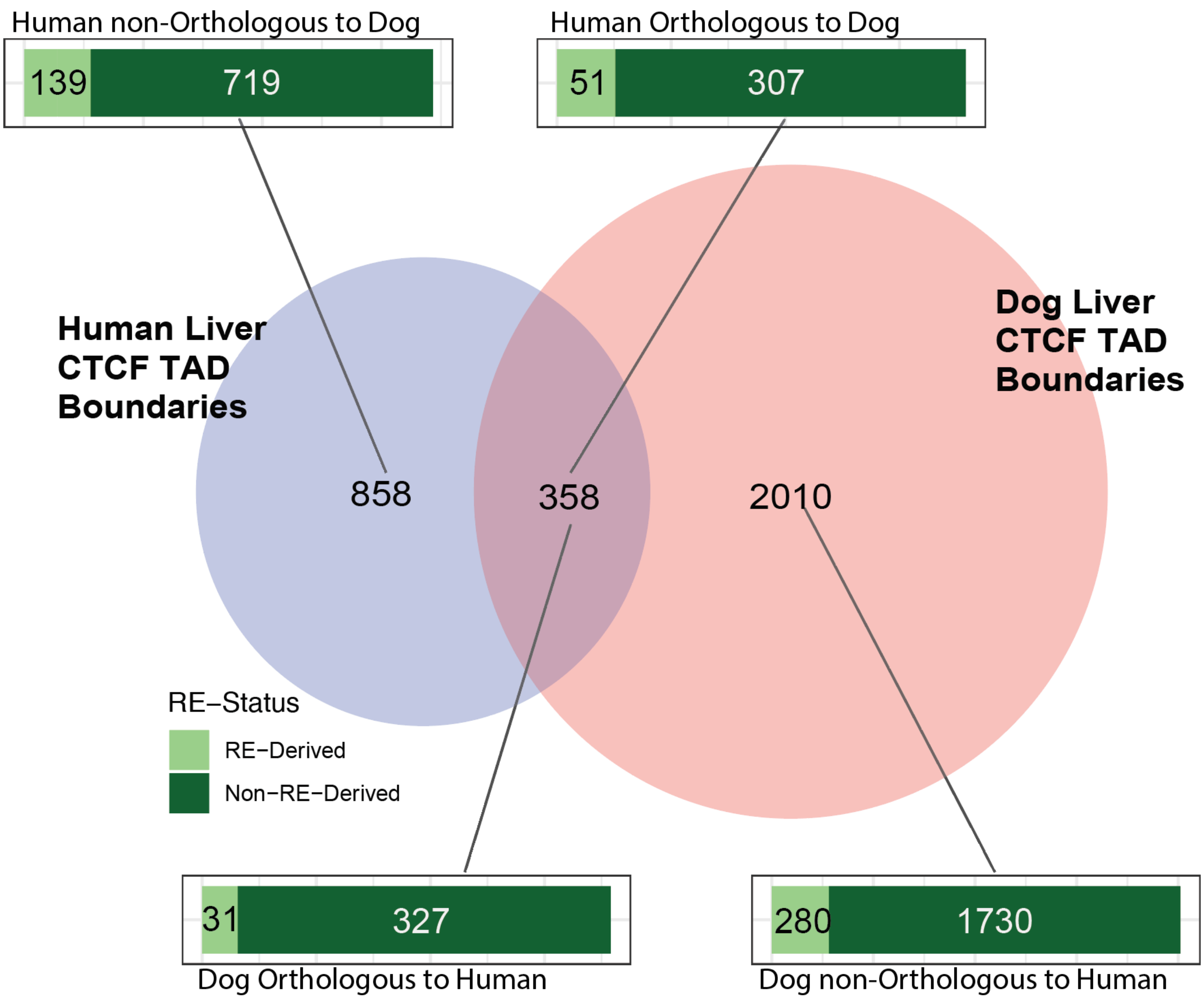

Dog liver TAD boundary data from Rudan et. al was lifted over from canFam3 to hg19 and compared with human liver data from Schmitt et. all to identify orthologous and non-orthologous CTCF TAD boundaries. The human liver data from Schmitt et. al was similarly lifted over to the canFam3 genome and compared to the dog liver data. These orthologous and non-orthologous TAD boundaries were then intersected with repeatMasker to identify RE-derived CTCF boundaries.

Supplementary Figure 4: MER57A1 Derived Loop Anchor

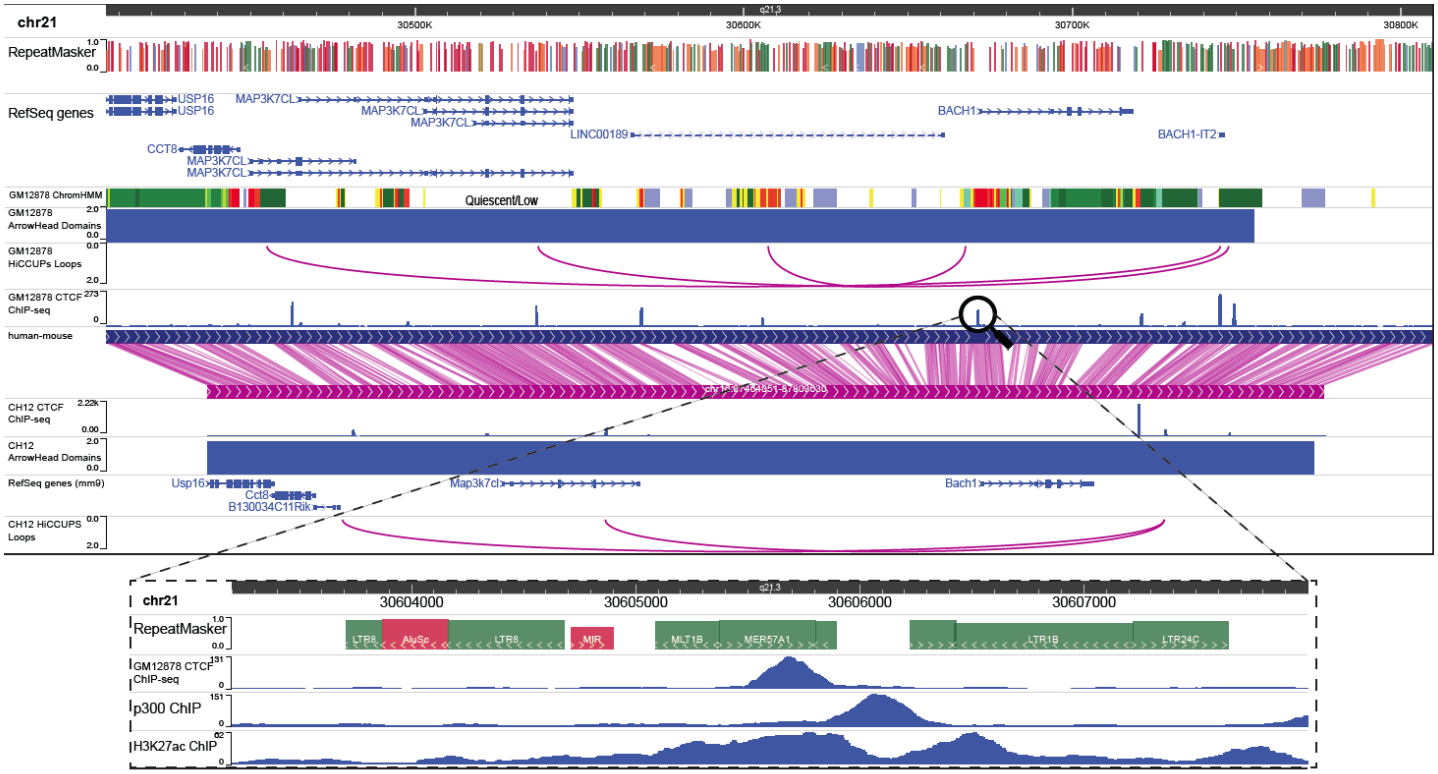

Genome browser screenshot displaying the MER57A1 candidate and the local histone, binding, 3D landscape in human (main top) and mouse (main bottom). Zoomed in view of the MER571A1 candidate with CTCF, p300, and H3K27ac ChIP-seq peaks (inset).

Supplementary Figure 5: LTR13 Derived Loop Anchor

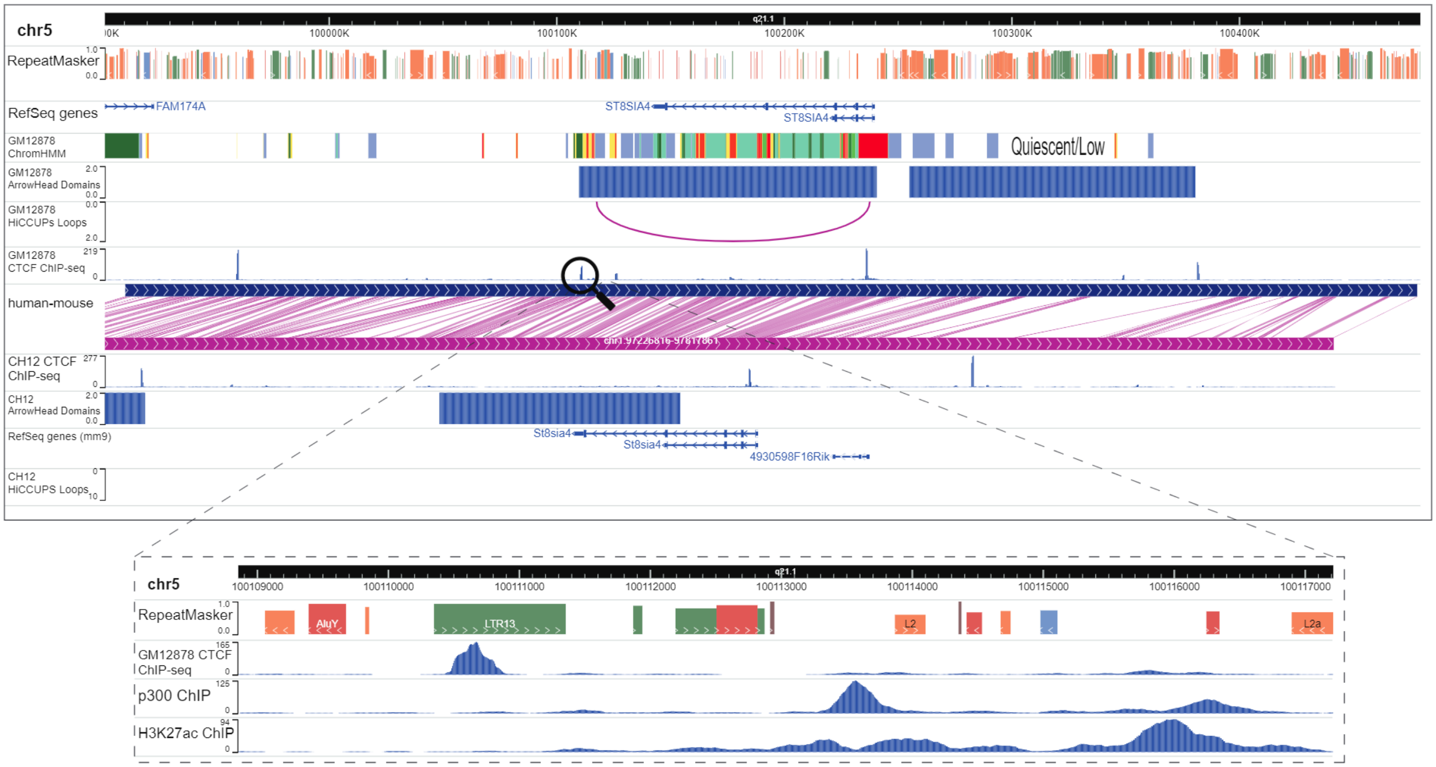

Genome browser screenshot displaying the LTR13 candidate and the local histone, binding, 3D landscape in human (main top) and mouse (main bottom). Zoomed in view of the LTR13 candidate with CTCF, p300, and H3K27ac ChIP-seq peaks (inset).

Supplementary Figure 6: L1MC1-KO specific deletions:

Primers, L2 sgRNA, R1 sgRNA, R2 sgRNA, L1MC1, **CTCF Motif**

Genomic Sequence:
TTCACGCTTCACTTGTCTGCgtacctcattcttcctagacgcaggacaagagctctggcaaaggcgctgcagccacagaggtttc
cggccggaaaaatcggcaccccagagatcccatgagaacgtcacgtacagcactgagacggagccctaatgaaaactgtggctct
gggtgataatgacgttactgtgggttcatcaactgtagcaaattc**gccctctgctggg**cgttgttggaaatgcagaggctctggg
gggtcaggtatgtgggagctctctgtacctttcttgtaatttgatgtgaacttaaagctgctccaaaaagataaaatcttgaaga
aaagcgaaaaaaaaaaaaaggaagagaggaaaaaatgacaggcagaaggtcacgcctggcccctcagtgcgcgtcctcaggcctc
tgctcccagctggggctggcaggtggtcggggggcacccagctcctccactccccacgcccaccacagctttcaaagttaggaa
cacaggcagggtGGAGGTGAAGTCCGAACAGA

Clone 1 (from forward primer):
TTCACGCTTCACTTGTCTGCgtacctcattcttcctagacgcaggacaagagctctggcaaaggcgctgcagccacagagg
tttccggccggaaaaatcggcaccccagagatcccatgagaacgtcacgtacagcactgagacggagccctaatgaaaact
gtggctctgggtgataatgacgtta...
.......................tgtgggagctctctgtacctttcttgtaatttgatgtgaacttaaagctgctccaaaaa
gataaaatcttgaagaaaagcgaaaaaaaaaaaaaggaagagaggaaaaaatgacaggcagaaggtcacgcctggcccctc
agtgcgcgtcctcaggcctctgctcccagctggggctggcaggtggtcggggggcacccagctcctccactccccacgcc
caccacagctttcaaagttaggaacacaggcagggtGGAGGTGAAGTCCGAACAGA

Clone 47 (Heterozygous, double knockout)

TTCACGCTTCACTTGTCTGCgtacctcattcttcctagacgcaggacaagagctctggcaaaggcgctgcagccacagagg
tttccggccggaaaaatcggcaccccagagatcccatgagaacgtcacgtacagcactgagacggagccctaatgaaaact
gtggctctgggtgataatgacgttactgtgggttcatcaactgtagcaaattc**gccctctgctggg**cgttgttggaaatgc
agaggctctgggggtcaggtatgtgggagctctctgtacctttcttgtaatttgatgtgaacttaaagctgctccaaaaa
gataaaatcttgaagaaaagcgaaaaaaaaaaaaaggaagagaggaaaaaatgacaggcagaaggtcacgcctggcccctc
agtgcgcgtcctcaggcctctgctcccagctggggctggcaggtggtcggggggcacccagctcctccactccccacgcc
caccacagctttcaaagttaggaacacaggcagggtGGAGGTGAAGTCCGAACAGA

TTCACGCTTCACTTGTCTGCgtacctcattcttcctagacgcaggacaagagctctggcaaaggcgctgcagccacagagg
tttccggccggaaaaatcggcaccccagagatcccatgagaacgtcacgtacagcactgagacggagccctaatgaaaact
gtggctctgggtgataatgacgttactgtgggttcatcaactgtagcaaattc**gccctctgctggg**cgttgttggaaatgc
agaggctctgggggtcaggtatgtgggagctctctgtacctttcttgtaatttgatgtgaacttaaagctgctccaaaaa
gataaaatcttgaagaaaagcgaaaaaaaaaaaaaggaagagaggaaaaaatgacaggcagaaggtcacgcctggcccctc
agtgcgcgtcctcaggcctctgctcccagctggggctggcaggtggtcggggggcacccagctcctccactccccacgcc
caccacagctttcaaagttaggaacacaggcagggtGGAGGTGAAGTCCGAACAGA

Deletions of the L1MC1-derived CTCF were confirmed via Sanger sequencing for two clones. The exact placement of the deletions is depicted in red.

Supplementary Figure 7: Virtual 4C Analysis of Downstream Enhancers

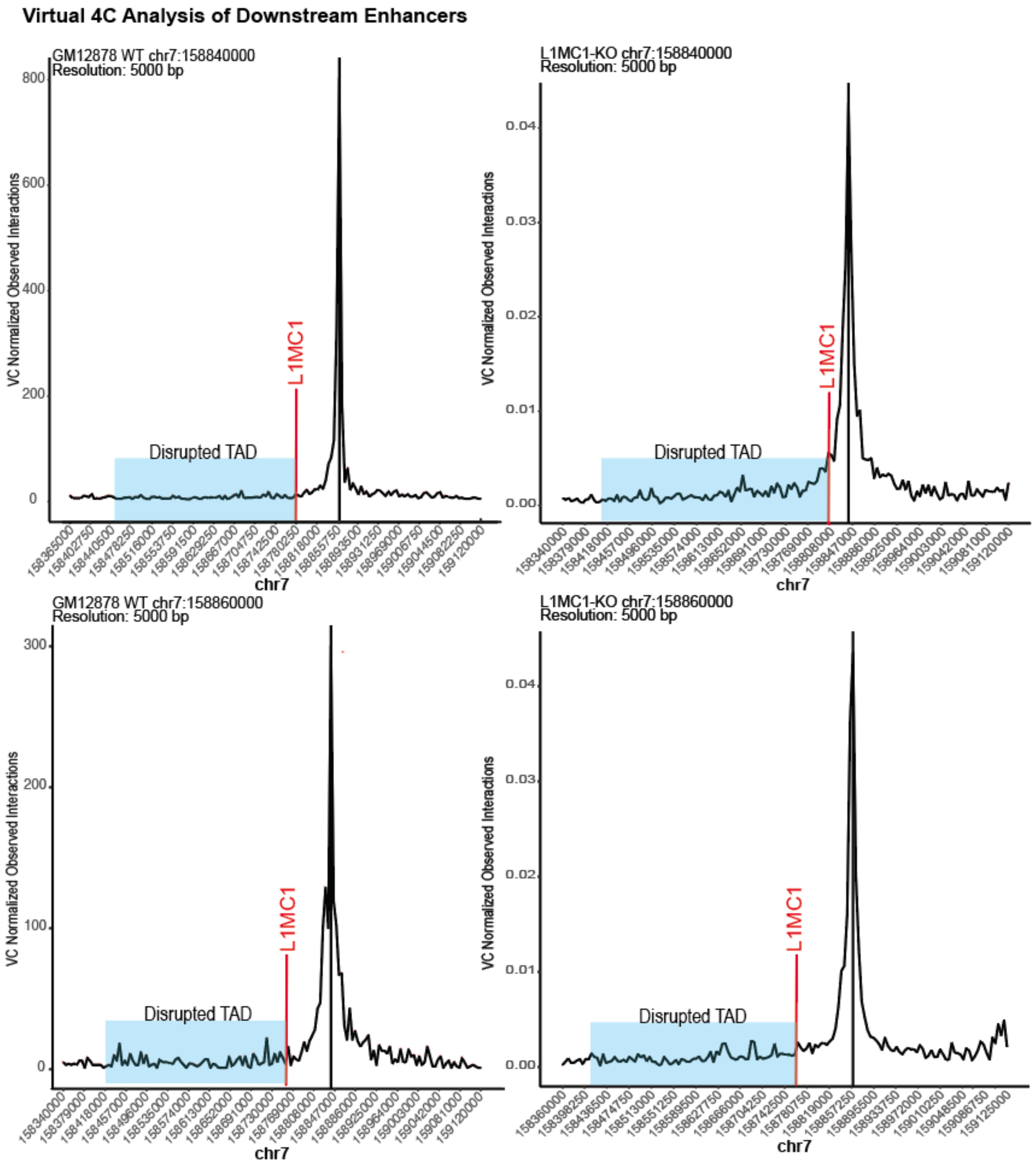

Virtual 4C analysis showing interactions between downstream enhancers and active domain in wildtype and L1MC1-KO lines.

Supplementary Table 1: Loop and TAD Call Data Sources

Data sources of loop and TAD calls used in all analyses.

Species	Structure	Assay	Cell Type	Link
Human	TADs	Hi-C	HSC_aml	https://www.biorxiv.org/content/10.1101/2020.04.18.047738v1
Human	TADs	Hi-C	HSC_aml	
Human	TADs	Hi-C	HSC_aml	
Human	TADs	Hi-C	HSC	
Human	TADs	Hi-C	HSC	
Human	TADs	Hi-C	HSC	
Human	Loops	Hi-C	HSC_aml	
Human	Loops	Hi-C	HSC_aml	
Human	Loops	Hi-C	HSC_aml	
Human	Loops	Hi-C	HSC	
Human	Loops	Hi-C	HSC	
Human	Loops	Hi-C	HSC	
Human	Loops	H3K4me2_ChIA-PET	CD4+ T Cells (CD4)	https://www.nature.com/articles/cr201215
Human	TADs	Hi-C	Cortex	https://www.nature.com/articles/nature11082
Human	TADs	Hi-C	hESC	
Human	TADs	Hi-C	IMR90	
Human	Loops	H3K27ac_HiChIP	GM12878	https://www.nature.com/articles/ng.3963
Human	Loops	H3K27ac_HiChIP	HCASMC	
Human	Loops	H3K27ac_HiChIP	K562	
Human	Loops	H3K27ac_HiChIP	MyLa	
Human	Loops	H3K27ac_HiChIP	Naive_T	
Human	Loops	H3K27ac_HiChIP	TH17	
Human	Loops	H3K27ac_HiChIP	TReg	
Mouse	Loops	H3K27ac_HiChIP	MES	
Human	Loops	CTCF_HiChIP	GM12878	https://www.nature.com/articles/nmeth.3999#Sec19
Mouse	Loops	CTCF_HiChIP	mESC	
Human	TADs	Hi-C	hESC	https://www.nature.com/articles/s41586-019-1812-0
Human	TADs	Hi-C	hESC	
Human	TADs	Hi-C	hESC	
Human	TADs	Hi-C	hESC	
Human	TADs	Hi-C	hESC	
Mouse	TADs	Hi-C	mESC	
Mouse	TADs	Hi-C	mESC	
Mouse	TADs	Hi-C	mESC	
Human	TADs	Hi-C	Lymphoma	https://www.nature.com/articles/s41588-018-0338-y

Human	TADs	Hi-C	Lymphoma	
Dog	TADs	Hi-C	Liver	https://www.ncbi.nlm.nih.gov/pmc/articles/PMC4542312/
Human	TADs	Hi-C	RPE1	https://www.ncbi.nlm.nih.gov/pmc/articles/PMC4978254/#d36e641
Human	TADs	Hi-C	RPE1	
Human	TADs	Hi-C	RPE1	
Mouse	TADs	Hi-C	PATSKI	
Mouse	TADs	Hi-C	mESC	
Rhesus Macaque	TADs	Hi-C	Fibroblasts	
Human	Loops	Hi-C	RPE1	
Human	Loops	Hi-C	RPE1	
Human	Loops	Hi-C	RPE1	
Mouse	Loops	Hi-C	PATSKI	
Rhesus Macaque	Loops	Hi-C	Fibroblasts	
Mouse	TADs	Hi-C	G1E-ER4	https://www.ncbi.nlm.nih.gov/pmc/articles/PMC5393350/
Mouse	TADs	Hi-C	G1E-ER4	
Human	TADs	Hi-C	Adrenal Cells (AD)	https://www.ncbi.nlm.nih.gov/pmc/articles/PMC5478386/
Human	TADs	Hi-C	Aorta (AO)	
Human	TADs	Hi-C	Bladder (BL)	
Human	TADs	Hi-C	Cortex (CO)	
Human	TADs	Hi-C	GM12878	
Human	TADs	Hi-C	hESC (H1)	
Human	TADs	Hi-C	Hippocampus (HC)	
Human	TADs	Hi-C	IMR90	
Human	TADs	Hi-C	Lung (LG)	
Human	TADs	Hi-C	Liver(LI)	
Human	TADs	Hi-C	Left Ventrical (LV)	
Human	TADs	Hi-C	Mesendoderm (MES)	
Human	TADs	Hi-C	Mesenchymal (MSC)	
Human	TADs	Hi-C	NPC	
Human	TADs	Hi-C	Ovary (OV)	
Human	TADs	Hi-C	Pancreas (PA)	
Human	TADs	Hi-C	Psoas Muscle (PO)	
Human	TADs	Hi-C	Right Ventrical (RV)	

Human	TADs	Hi-C	Small Bowel (SB)	
Human	TADs	Hi-C	Spleen (SX)	
Human	TADs	Hi-C	Trophoblast -like (TRO)	
Human	Loops	Hi-C	THP-1_pma	https://www.ncbi.nlm.nih.gov/pmc/articles/PMC5610110/
Human	Loops	Hi-C	THP-1	
Mouse	TADs	Hi-C	AML12	https://www.ncbi.nlm.nih.gov/pmc/articles/PMC5793783/
Mouse	Loops	Hi-C	mESC	https://www.ncbi.nlm.nih.gov/pmc/articles/PMC6327227/
Mouse	Loops	Hi-C	NSC	
Human	TADs	Hi-C	LCL	https://www.ncbi.nlm.nih.gov/pmc/articles/PMC6883528/
Human	Loops	CTCF_ChIA-PET	Consensus	
Mouse	TADs	Hi-C	CH12-LX	https://www.ncbi.nlm.nih.gov/pubmed/25497547
Human	TADs	Hi-C	GM12878	
Human	TADs	Hi-C	HeLa	
Human	TADs	Hi-C	HMEC	
Human	TADs	Hi-C	HUVEC	
Human	TADs	Hi-C	IMR90	
Human	TADs	Hi-C	K562	
Human	TADs	Hi-C	KBM7	
Human	TADs	Hi-C	NHEK	
Human	Loops	Hi-C	GM12878	
Human	Loops	Hi-C	HeLa	
Human	Loops	Hi-C	HMEC	
Human	Loops	Hi-C	HUVEC	
Human	Loops	Hi-C	IMR90	
Human	Loops	Hi-C	K562	
Human	Loops	Hi-C	KBM7	
Human	Loops	Hi-C	NHEK	
Mouse	Loops	Hi-C	CH12-LX	
Human	TADs	Hi-C	HAP1	https://www.pnas.org/content/112/47/E6456.long
Human	Loops	Hi-C	HAP1	
Human	Loops	Hi-C	HSC	https://www.sciencedirect.com/science/article/pii/S1097276520302604

Supplementary Table 2: L1MC1-KO1 HiC^2 Library Statistics

Sequenced Read Pairs	101,630,014
Normal Paired	90,448,638 (89.00%)
Chimeric Paired	4,407,936 (4.34%)
Chimeric Ambiguous	3,513,938 (3.46%)
Unmapped	3,259,502 (3.21%)
Ligation Motif Present	29,354,231 (28.88%)
Alignable (Normal+Chimeric Paired)	94,856,574 (93.34%)
Unique Reads	28,973,498 (28.51%)
PCR Duplicates	65,831,160 (64.78%)
Optical Duplicates	51,916 (0.05%)
Library Complexity Estimate	30,299,572
Intra-fragment Reads	325,061 (0.32% / 1.12%)
Below MAPQ Threshold	19,264,376 (18.96% / 66.49%)
Hi-C Contacts	9,384,061 (9.23% / 32.39%)
Ligation Motif Present	2,054,781 (2.02% / 7.09%)
3' Bias (Long Range)	69% - 31%
Pair Type %(L-I-O-R)	25% - 25% - 25% - 25%
Inter-chromosomal	2,431,679 (2.39% / 8.39%)
Intra-chromosomal	6,952,382 (6.84% / 24.00%)
Short Range (<20Kb)	2,615,029 (2.57% / 9.03%)
Long Range (>20Kb)	4,337,335 (4.27% / 14.97%)

Supplementary Table 3: L1MC1-KO47 HiC^2 Library Statistics

Sequenced Read Pairs	29,681,164
Normal Paired	26,419,325 (89.01%)
Chimeric Paired	1,505,030 (5.07%)
Chimeric Ambiguous	835,886 (2.82%)
Unmapped	920,923 (3.10%)
Ligation Motif Present	8,772,298 (29.56%)
Alignable (Normal+Chimeric Paired)	27,924,355 (94.08%)
Unique Reads	15,224,433 (51.29%)
PCR Duplicates	12,685,207 (42.74%)
Optical Duplicates	14,715 (0.05%)
Library Complexity Estimate	20,445,316
Intra-fragment Reads	166,830 (0.56% / 1.10%)
Below MAPQ Threshold	6,958,217 (23.44% / 45.70%)
Hi-C Contacts	8,099,386 (27.29% / 53.20%)
Ligation Motif Present	2,199,042 (7.41% / 14.44%)
3' Bias (Long Range)	71% - 29%
Pair Type %(L-I-O-R)	25% - 25% - 25% - 25%
Inter-chromosomal	1,929,082 (6.50% / 12.67%)
Intra-chromosomal	6,170,304 (20.79% / 40.53%)
Short Range (<20Kb)	2,227,463 (7.50% / 14.63%)
Long Range (>20Kb)	3,942,828 (13.28% / 25.90%)

Supplementary Table 4: GM12878 Wild Type HiC^2 Library Statistics

Normal Paired	269,686,068 (83.48%)
Chimeric Paired	16,914,983 (5.24%)
Chimeric Ambiguous	4,759,644 (1.47%)
Unmapped	31,677,013 (9.81%)
Ligation Motif Present	84,083,628 (26.03%)
Alignable (Normal+Chimeric Paired)	286,601,051 (88.72%)
Unique Reads	65,938,196 (20.41%)
PCR Duplicates	220,546,478 (68.27%)
Optical Duplicates	116,377 (0.04%)
Library Complexity Estimate	66,859,184
Intra-fragment Reads	799,217 (0.25% / 1.21%)
Below MAPQ Threshold	21,862,935 (6.77% / 33.16%)
Hi-C Contacts	43,276,044 (13.40% / 65.63%)
Ligation Motif Present	11,358,479 (3.52% / 17.23%)
3' Bias (Long Range)	71% - 29%
Pair Type %(L-I-O-R)	25% - 25% - 25% - 25%
Inter-chromosomal	7,210,469 (2.23% / 10.94%)
Intra-chromosomal	36,065,575 (11.16% / 54.70%)
Short Range (<20Kb)	15,110,002 (4.68% / 22.92%)
Long Range (>20Kb)	20,955,480 (6.49% / 31.78%)
