# Supplementary material for: Widespread contribution of transposable elements to the rewiring of mammalian 3D genomes
Source: Nat Commun. 2023 Feb 6;14:634. doi: 10.1038/s41467-023-36364-9 (PMC9902604; doi:10.1038/s41467-023-36364-9)
Supplement: Supplementary file 3 — Reporting Summary [file 41467_2023_36364_MOESM3_ESM.pdf]

Corresponding author(s): Ting Wang

Last updated by author(s): 01/13/2023

Reporting Summary

Nature Portfolio wishes to improve the reproducibility of the work that we publish. This form provides structure for consistency and transparency in reporting. For further information on Nature Portfolio policies, see our Editorial Policies and the Editorial Policy Checklist.

Statistics

For all statistical analyses, confirm that the following items are present in the figure legend, table legend, main text, or Methods section.

n/a	Confirmed	
☐	☒	The exact sample size (*n*) for each experimental group/condition, given as a discrete number and unit of measurement
☐	☒	A statement on whether measurements were taken from distinct samples or whether the same sample was measured repeatedly
☐	☒	The statistical test(s) used AND whether they are one- or two-sided<br>*Only common tests should be described solely by name; describe more complex techniques in the Methods section.*
☒	☐	A description of all covariates tested
☒	☐	A description of any assumptions or corrections, such as tests of normality and adjustment for multiple comparisons
☒	☐	A full description of the statistical parameters including central tendency (e.g. means) or other basic estimates (e.g. regression coefficient) AND variation (e.g. standard deviation) or associated estimates of uncertainty (e.g. confidence intervals)
☒	☐	For null hypothesis testing, the test statistic (e.g. *F*, *t*, *r*) with confidence intervals, effect sizes, degrees of freedom and *P* value noted<br>*Give P values as exact values whenever suitable.*
☒	☐	For Bayesian analysis, information on the choice of priors and Markov chain Monte Carlo settings
☒	☐	For hierarchical and complex designs, identification of the appropriate level for tests and full reporting of outcomes
☒	☐	Estimates of effect sizes (e.g. Cohen's *d*, Pearson's *r*), indicating how they were calculated

Our web collection on statistics for biologists contains articles on many of the points above.

Software and code

Policy information about availability of computer code

Data collection	No software was used
Data analysis	HiC and HiC^2 data were analyzed according to Rao et. al, 2014, via the juicer software (version 1.9.9). RNA-seq data was aligned with STAR 2.5.4b and analyzed using edgeR v3.26.8. ATAC-seq was aligned using AIAP(Liu et. al 2021), and was analyzed using DiffBind v2.14.

For manuscripts utilizing custom algorithms or software that are central to the research but not yet described in published literature, software must be made available to editors and reviewers. We strongly encourage code deposition in a community repository (e.g. GitHub). See the Nature Portfolio guidelines for submitting code & software for further information.

Data

Policy information about availability of data

All manuscripts must include a data availability statement. This statement should provide the following information, where applicable:

- Accession codes, unique identifiers, or web links for publicly available datasets
- A description of any restrictions on data availability
- For clinical datasets or third party data, please ensure that the statement adheres to our policy

Hi-C2, RNA-seq, and ATAC-seq data generated in this study has been deposited in the Gene Expression Omnibus (GEO) database under accession code GSE222526 [https://www.ncbi.nlm.nih.gov/geo/query/acc.cgi?acc=GSE222526].

Human research participants

Policy information about studies involving human research participants and Sex and Gender in Research.

Reporting on sex and gender	N/A
Population characteristics	N/A
Recruitment	N/A
Ethics oversight	N/A

Note that full information on the approval of the study protocol must also be provided in the manuscript.

Field-specific reporting

Please select the one below that is the best fit for your research. If you are not sure, read the appropriate sections before making your selection.

- [x] Life sciences
- [ ] Behavioural & social sciences
- [ ] Ecological, evolutionary & environmental sciences

For a reference copy of the document with all sections, see nature.com/documents/nr-reporting-summary-flat.pdf

Life sciences study design

All studies must disclose on these points even when the disclosure is negative.

Sample size	Due to the well-catalogued nature of the GM12878 line as a control, the two knockout lines are sufficient to see a difference in chromatin structure. One technical replicate is a field standard for CRISPR.
Data exclusions	No data were excluded.
Replication	We were able to replicate the impacts of CRISPR deletion of the targeted L1MC1 element in one additional clone. This involved a single independent replication of CRISPR, HiC, RNA-seq, and ATAC-seq.
Randomization	The cells were split, and half of the cells were assigned to remain wildtype, and the other half went on to have the CRISPR treatment. The group of cells receiving treatment was chosen at random.
Blinding	Blinding was not performed in this study, as the wild type control cells underwent HiC at a later time, during revision.

Reporting for specific materials, systems and methods

We require information from authors about some types of materials, experimental systems and methods used in many studies. Here, indicate whether each material, system or method listed is relevant to your study. If you are not sure if a list item applies to your research, read the appropriate section before selecting a response.

Materials & experimental systems

n/a	Involved in the study	
x		Antibodies
	x	Eukaryotic cell lines
x		Palaeontology and archaeology
x		Animals and other organisms
x		Clinical data
x		Dual use research of concern

Methods

n/a	Involved in the study	
x		ChIP-seq
x		Flow cytometry
x		MRI-based neuroimaging

Eukaryotic cell lines

Policy information about cell lines and Sex and Gender in Research

Cell line source(s)	GM12878 cells (female) are human B-lymphocyte cells, and were obtained from The Coriell Institute for Medical Research
Authentication	Cell Lines were not authenticated.
Mycoplasma contamination	Cell lines were not tested for mycoplasma contamination.
Commonly misidentified lines (See ICLAC register)	No commonly misidentified cell lines were used in this study.
